# Influence of tamoxifen on ocular morphology and function

**DOI:** 10.1093/oncolo/oyaf103

**Published:** 2025-05-16

**Authors:** Piotr Strzalkowski, Alicja Strzalkowska, Maximiliane von der Ahe, Ann-Kathrin Ozga, Svjetlana Mohrmann, Rainer Guthoff, Gerd Geerling, Kristina Spaniol

**Affiliations:** Department of Ophthalmology - Medical Faculty and University Hospital Düsseldorf - Heinrich Heine University Düsseldorf, 40225 Düsseldorf, Germany; Department of Ophthalmology - Medical Faculty and University Hospital Düsseldorf - Heinrich Heine University Düsseldorf, 40225 Düsseldorf, Germany; Department of Ophthalmology - Medical Faculty and University Hospital Düsseldorf - Heinrich Heine University Düsseldorf, 40225 Düsseldorf, Germany; Institute of Medical Biometry and Epidemiology, University Medical Center Hamburg Eppendorf, 20251 Hamburg, Germany; Department of Gynecology - Medical Faculty and University Hospital Düsseldorf - Heinrich Heine University Düsseldorf, 40225 Düsseldorf, Germany; Department of Ophthalmology - Medical Faculty and University Hospital Düsseldorf - Heinrich Heine University Düsseldorf, 40225 Düsseldorf, Germany; Department of Ophthalmology - Medical Faculty and University Hospital Düsseldorf - Heinrich Heine University Düsseldorf, 40225 Düsseldorf, Germany; Department of Ophthalmology - Medical Faculty and University Hospital Düsseldorf - Heinrich Heine University Düsseldorf, 40225 Düsseldorf, Germany

**Keywords:** tamoxifen, visual field, corneal thickness, glaucoma risk, contrast sensitivity

## Abstract

**Background:**

Tamoxifen, a commonly used selective estrogen receptor antagonist for adjuvant therapy in hormone receptor-positive breast cancer, can cause ocular side effects due to estrogen receptors in ocular structures. This prospective study explores its impact on ocular morphology and function.

**Materials and methods:**

This prospective, non-interventional, cross-sectional study explores the effects of tamoxifen on ocular morphology, visual acuity, and quality. Examinations included objective refractometry, visual acuity, contrast sensitivity, color vision, visual field tests, corneal topography, optical coherence tomography, autofluorescence, and slit lamp biomicroscopy. Participants completed a health questionnaire. Statistical analysis, using linear or logistic (mixed) regression, compared tamoxifen’s effects to a control group.

**Results:**

A total of 102 patients were included: 90 in the study group and 12 controls. The duration of tamoxifen therapy showed a clinically relevant impact on the visual field, with a mean loss variance change of 0.045 dB² per week (95% CI [0.012 dB²; 0.079 dB²]). Corneal thickness decreased by −0.12 μm per week (95% CI [−0.20 μm; −0.031 μm]) with longer therapy duration. Fourteen patients received additional aromatase inhibitors, and this group showed clinically significant reductions in both visual acuity and contrast vision. No correlation was observed between tamoxifen intake and visual acuity, contrast vision, color vision, mean defect in visual fields, retinal thickness, macular morphology, or slit-lamp biomicroscopy findings.

**Conclusion:**

According to this study, tamoxifen intake influences corneal thickness and perimetry, which are two parameters associated with glaucoma development. Regular ophthalmological glaucoma controls could be useful in tamoxifen patients, especially if glaucoma is already known.

**Implications for practice**

This study highlights the potential impact of tamoxifen therapy on corneal thickness and visual field parameters, both of which are associated with risk of glaucoma. For patients undergoing tamoxifen treatment, regular ophthalmic examinations, including glaucoma screening, are recommended, especially for those with pre-existing glaucoma. Monitoring these changes could help reduce the risk of glaucoma progression and preserve visual function.

## Introduction

Breast cancer is the most common malignancy among women, with 67%-81% of cases being estrogen receptor positive (ER+).^1^ For decades, tamoxifen has been the gold standard for endocrine treatment of all stages of ER + breast cancer.^2^ The therapy lasts initially 5 years, with an option to extend it as adjuvant therapy (EAT) for another 5 years, or until a recurrence occurs.

Beginning in the mid-1980s, concerns arose about tamoxifen side effects, such as enhancing the growth of endometrial cancer in laboratory settings^3,4^ and was predicted to increase the risk of endometrial cancer in women as well.^4^ Moreover, ocular side effects have also been described, such as preretinal crystalline deposits with macular edema,^5^ cataract,^6^ keratopathy,^7^ dry eye,^8^ retinopathy^9^ and optic neuritis. This is due to the presence of ER + tissue within the eye,^10^ and tamoxifen has been detected in both vitreous and aqueous humors.^11^

In ER+ breast cancer, aromatase inhibitors are also commonly used. There is often an increased expression of aromatase in the endothelial cells of the breast, leading to enhanced estrogen production and stimulation of tumor growth. Aromatase inhibitors can also provoke ocular side effects, such as dry eye, retinal hemorrhages, visual disturbances, macular edema, uveitis, central artery occlusions, and vitreoretinal traction.^12^

Despite these case descriptions, data on the ocular side effects of tamoxifen and aromatase inhibitors are still insufficient. The aim of the present work was to examine the impact of tamoxifen and aromatase inhibitors on ocular structures, visual acuity, and visual quality through comprehensive ophthalmological examinations.

### Materials and methods

As part of this prospective, non-interventional cross-sectional explorative study (Registration ID: 2015104462, Ethics Number 5284R), 102 patients were included between May 1, 2016, and May 1, 2017, and examined at the University Eye Clinic Düsseldorf. Following examinations were performed in a neutral pupil position: objective refractometry (ARK-560A®, Oculus/Nidek, Wetzlar, Germany), visual acuity determination, contrast sensitivity test (MARS Letter Contrast Sensitivity Test, The Mars Perceptrix Corporation, Chappaqua, New York, USA), color vision test (Lanthony’s Desaturated 15 Hue Test, Luneau, Chartres and Paris, France, Visual field-testing Octopus 101, Haag-Streit, Wedel, Germany), corneal topography (Pentacam HR, Oculus, Wetzlar, Germany), optical coherence tomography (Spectralis, Heidelberg Engineering, Heidelberg, Germany), autofluorescence examination (Spectralis, Heidelberg Engineering, Heidelberg, Germany), examination of the anterior and posterior segments of the eye using slit lamp biomicroscopy (Table 1). In addition to height and weight, following health data information were collected using a questionnaire: amblyopia, glaucoma, dry eye, hypertension, hypothyroidism, hyperthyroidism, diabetes mellitus, nicotine consumption, alcohol consumption, GnRH analogs, therapy with aromatase inhibitors, chemotherapy, and antibody therapy (Table 2).

**Table 1.** Clinical ophthalmological investigations.

Test	Target parameter	Clinical relevance
Objective refractometry	Measure the refractive error of the patient´s eyes	Provides information if glasses are required to gain full visual acuity
Visual acuity testing	Measures the spatial resolution of the eye	Determines the best visual acuity and thus shows a potential visual acuity reduction
Contrast sensitivity testing	Measures the least bright contrast which can be discriminated by the patient	Multiple ocular diseases cause a reduction in contrast sensitivity; sometimes before visual acuity is affected
Color vision testing	Measures the ability to discriminate colors	Certain diseases of the retina and optic nerve cause a deterioration in color vision discrimination
Visual field testing	Measures the least bright light stimulus recognized by the patient at a certain point of the central visual field.	Diseases of the macula and the optic nerve-—as glaucoma-—cause a loss of sensitivity in the visual field test
Mean defect	Parameter to analyze the visual field	Measures the sensitivity loss compared a healthy population. Useful to determine the progression of visual field defects. Cannot discriminate between localized and diffuse defects
Loss variance	Parameter to analyze the visual field	Measures the variability of the sensitivity loss at different points of the visual field and can thus help to discriminate between diffuse (unspecific) and localized (glaucomatous) changes.
Corneal topography	Measures the anatomy of the corneal surface and back as well as the corneal thickness	Different ocular pathologies as well as local and systemic medication can cause changes in corneal anatomy and thickness
Optical coherence tomography of the retina	Measures the thickness of the different retinal layers based on coherent light beams	Provides information about the integrity of the different retinal layers
Autofluorescence measurement	Measures the retinal fluorescence with a blue laser light	Provides information about the integrity of the retinal pigment epithelium
Slit lamp investigation	Biomicroscopy of the anterior and posterior segments of the eye (from the eye lids to the retina)	Fundamental ophthalmological investigation providing information about ocular pathologies such as signs of dry eye, corneal opacities, cataract and vitreal or retinal abnormalities

**Table 2.** Patient’s characteristics for the study group..

	Tamoxifen (*n* = 90)	Control group (*n* = 12)
Age in years	Median 51.5 IQR 10.5 Mean ± SD 53.4 ± 9.1 Minimum 32 Maximum 78	Median 50.5 IQR 6.8 Mean ± SD 51.1 ± 7.4 Minimum 35 Maximum 65
Tamoxifen intake duration in weeks	Median 76.14 IQR 114.96 Mean ± SD 98.5 ± 86.2 Minimum 1 Maximum 344	-
Amblyopia	2 (2.2%)	0
Dry eye	34 (37.8%)	3 (25.0%)
Hyperthyroidism	2 (2.2%)	0
Nicotine consumption	13 (14.4%)	2 (16.7%)
Alcohol consumption	12 (13.3%)	0
GnRH analogs	0	1 (8.3%)
Antibody therapy	3 (3.3%)	1 (8.3%)

The parameters amblyopia, dry eye, hyperthyroidism, nicotine abuse, alcohol abuse, GnRH analogs and antibody therapy were analyzed as dichotomous parameters (yes/no). Alcohol consume was defined as drinking at least one glass of beer or wine each day.

Abbreviaitons: GnRH, gonadotropine releasing hormone; IQR, interquartile range, SD, standard deviation;

The parameters amblyopia, dry eye, hyperthyroidism, nicotine consumption, alcohol consumption, GnRH analogs and antibody therapy were analyzed as dichotomous parameters (yes/no). Alcohol consumption was defined as drinking at least one glass of beer or wine each day.

An experienced ophthalmologist performed a slit lamp biomicroscopy and fundoscopy to evaluate the anterior and posterior segments of the eye, respectively.

### Statistical analysis

For the description of patient characteristics categorical data are summarized via absolute and relative frequencies and continuous data are summarized as mean values with standard deviations or as median values with interquartile ranges, as appropriate.

To analyze the influence of tamoxifen therapy (Tx) vs control we performed univariable and multivariable linear or logistic (mixed) regression, as appropriate. Mixed models were used in case two values per patient (ie, one for each eye) were given and hence a random intercept for the patient was included in the model. Variables that showed a relevant influence in the univariable models were included in the multivariable regression models. The following dependent variables were considered: average loss variance, average mean defect, corneal thickness, average best-corrected visual acuity, contrast sensitivity test, Color Confusion Index, retinal thickness, and blepharitis. As independent variables we considered: Tx (tamoxifen therapy (vs control)), age, hypertension, diabetes mellitus, aromatase inhibitors, glaucoma). Model assumptions were checked via histograms of residuals. Missing values were not imputed and no adjustment for multiple testing was conducted. All *P*-values are descriptive due to the explorative character of the study.

## Results

The study included data from 102 patients: 90 on Tx and 12 patients diagnosed with hormone receptor positive breast cancer but without Tx (control). Among the Tx group, 13 patients also used an aromatase inhibitor during treatment (Table 2).

Data from the questionnaire on amblyopia, glaucoma, dry eye, hyperthyroidism, nicotine consumption, alcohol consumption, GnRH analogs, and antibody therapy are shown in Table 2. No clinically relevant differences were seen.

### Dry eye

Reported prevalence of dry eye was equal in all groups, as 32 out of 90 (35.6%) Tx vs 5 out of 12 (41.7%) control patients stated to suffer from dry eyes. Six out of 13 patients who received aromatase inhibitors suffered from dry eyes (46.2%).

### Visual field

Visual field testing is a common ophthalmological investigation to analyze defects related to retinal pathologies or optic nerve diseases such as glaucoma. Herein, loss variance allows us to discriminate between localized defects as seen in (early) glaucoma, and diffuse defects, which are for example caused by cataracts. Mean defect shows sensitivity loss compared to the healthy population and is used to recognize glaucoma progression.

### Loss variance

**Figure 1:**
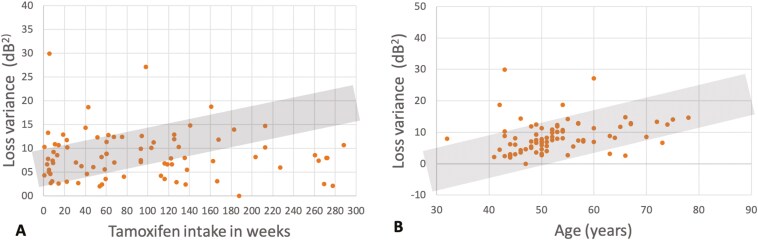
Influence of tamoxifen intake and age on the loss variance in the visual field examination. Visual field testing is a common instrument to analyze diseases of the retina and the optic nerve as nerve fiber loss leads to visual field defects. Herein, loss variance allows to discriminate between localized defects as seen in (early) glaucoma and diffuse defects as for example seen in case of opacities of cornea and lens. (A) The duration of Tx intake seemed to have a small influence with a mean change in loss variance of 0.045 dB² per week (95% CI [0.012 dB² per week; 0.079 dB² per week]; univariable analysis). B: Regarding age, the univariable analysis showed a mean increase of 0.39 dB² per year (95% CI [0.11 dB² per year; 0.67 dB² per year], *P* = .008). In the multivariable analysis (with other independent variable: Tamoxifen treatment vs control) an estimated increase in mean of 0.38 dB² per year (95% CI [0.10 dB² per year; 0.67 dB² per year]) was observed.

The average loss variance was 10.1 ± 13.7 dB². Overall, Tx intake did not relevantly affect loss variance, with *P* = .53 (2.57, 95% CI [−5.51 dB²; 10.66 dB²]) in the univariable analysis and *P* = .44 (0.03, 95% CI [−0.05 dB²; 0.10 dB²]) in the multivariate analysis (with further independent variables: age, diabetes mellitus, other aromatase inhibitors). However, the duration of Tx intake seemed to have a small influence with a mean change in loss variance of 0.045 dB² per week (95% CI [0.012 dB² per week; 0.079 dB² per week]; univariable analysis) (Figure 1A).

Changes in loss variance were related to age. The univariable analysis showed a mean increase of 0.39 dB² per year (95% CI [0.11 dB² per year; 0.67 dB² per year], *P* = 0.008). In the multivariate analysis (with other independent variable: Tx vs control) an estimated increase in mean of 0.38 dB² per year (95% CI [0.10 dB² per year; 0.67 dB² per year]) was observed (Figure 1B).

In patients with glaucoma, loss variance worsened relevantly as seen in the univariable analysis, with an estimated effect of 25.98 dB². This estimate is imprecise, with a confidence interval of [95% CI: 15.06 dB², 36.89 dB²]. Because of this imprecision estimation of the effect in a multivariable model was not possible.

### Mean defect

The average mean defect in the Tx group was −6.79 ± 3.12 dB. Tx intake did not relevantly affect the mean defect, with *P*-values of 0.14 (1.23 dB, 95% CI [−0.42 dB; 3.00 dB]) in the univariable analysis and 0.19 (1.13 dB, 95% CI [−0.55 dB; 2.81 dB]) in the multivariable analysis (other independent variable in the model: glaucoma). However, Tx patients with a previous glaucoma diagnosis (*n* = 5) had a worse mean defect compared to control patients (effect in univariable analysis 3.01 dB (95% CI [0.50 dB; 5.52 dB]), *P* = 0.019; effect in multivariable analysis 2.87 dB (95% CI [0.36 dB; 5.38 dB]), *P* = 0.026; other independent variable in the model: Tx). However, the confidence interval was wide, indicating an imprecise estimation.

### Pachymetry

The corneal thickness is measured by pachymetry and influenced by several ophthalmological diseases but also by certain eye drops or systemic diseases. A thin cornea leads to lower values when measuring the intraocular pressure and vice versa. A significantly swollen cornea leads to visual acuity deterioration.

**Figure 2.**
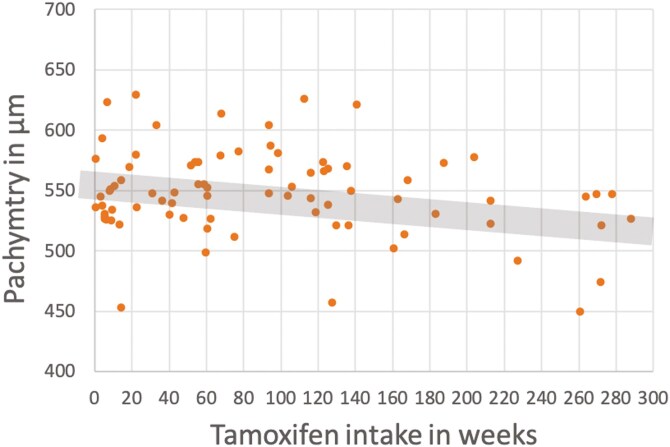
Influence of tamoxifen intake on corneal thickness. The corneal thickness is measured by pachymetry and influenced by several ophthalmological diseases but also by certain eye drops or systemic diseases. A thin cornea leads to lower values when measuring the intraocular pressure and vice versa. A significantly swollen cornea leads to visual acuity deterioration. A longer tamoxifen intake was associated with a thinner cornea (−0.12 μm per week (95% CI [−0.20 μm per week; −0.031 μm per week]), *P* = .008; univariable analysis).

Overall, Tx intake did seem not to influence corneal thickness (*P* = 0.73; -3.8 µm, 95% CI [-25.85 µm; 18.25 µm]; univariable analysis). However, a longer Tx intake was associated with a thinner cornea (−0.12 μm per week (95% CI [−0.20 μm per week; −0.031 μm per week]), *P* = 0.008; univariable analysis) (Figure 2).

### Visual acuity

The visual acuity describes the ability of the eye to resolve fine details expressed as the reciprocal of the minimum angular separation of two lines. It therefore shows the individual's sharpness of vision after correcting for refractive errors.

The average best-corrected visual acuity (BCVA) for the entire patient group was 0.02 ± 0.18 logMAR. Tx did not have a relevant effect on visual acuity, with *P*-values of 0.83 (-0.01 log MAR units, 95% CI [−0.11 log MAR units; 0.08 log MAR units]) in the univariable analyses and 0.56 (−0.03 log MAR units, 95% CI [-0.12 log MAR units; 0.07 log MAR units]) in the multivariable analysis (other independent variables in the model: age, diabetes mellitus, aromatase inhibitors).

Age was related to BCVA (*P* = 0.0004; 0.006, 95% CI [0.0004; 0.009]; per year; univariable analysis). Data showed a very small annual BCVA decrease (0.0059 log MAR units per year).

Four patients had diabetes mellitus at the time of the examination. Univariable analysis showed difference between diabetes mellitus yes vs no of 0.187logMAR units, (95% CI [0.034 logMAR units; 0.341 logMAR units]) on visual acuity (*P*-value of.017).

Additionally, using aromatase inhibitors alongside Tx showed an influence on visual acuity. The univariable analysis showed a change of 0.126 logMAR (95% CI [0.040 logMAR units; 0.211 logMAR units]; *P*-value of.004).

In the multivariable analysis, the effect of aromatase inhibitors showed a small, estimated decline in visual acuity of 0.098 logMAR (95% CI [0.004 logMAR units; 0.193 logMAR units]; *P*-value of.041; other independent variables in the model: Tx, age, diabetes mellitus).

### Contrast sensitivity

Contrast sensitivity is defined as the ability to detect fine differences in shadings. It is for example important to detect margins of objects in surroundings with reduced lighting. Contrast sensitivity is reduced by several ophthalmological disorders, as well of the anterior as of the posterior segments of the eye.

In the contrast sensitivity test, the average value for all patients was 1.67 ± 0.09 log Contrast Sensitivity (logCS). Tx did not relevantly affect contrast sensitivity, with *P*-value of 0.29 (-0.03 logCS, 95% CI [−0.09 logCS; 0.03 logCS]) in the univariable analysis and 0.55 (−0.02 logCS, 95% CI [−0.07 logCS; 0.04 logCS]) in the multivariable analysis (other independent variables in the model: age, diabetes mellitus, aromatase inhibitors).

For the influence of age on contrast sensitivity following small effects were seen: the univariable analysis found a decline of about −0.0044 logCS per year (CI [−0.0063 logCS; −0.0025 logCS]; *P*-value of 0.00001; the multivariable analysis also showed a decline of -0.0025 logCS per year (CI [−0.0047 logCS; −0.0003 logCS]; *P* = .026; other independent variables in the model: Tx, diabetes mellitus, aromatase inhibitors).

Simultaneous therapy with aromatase inhibitors had a negative impact on contrast sensitivity. The univariable analysis estimated a decrease of −0.109 logCS (CI [−0.060; −0.159]; *P* = .00003), and the multivariable analysis estimated a decrease of −0.082 logCS (CI [−0.137; −0.028]; *P* = .00346; other independent variables in the model: Tx, diabetes mellitus, age).

### Color vision

Color Vision is enabled by the retinal cones. Color vision deficiencies can be inherited or acquired by retinal or optic nerve diseases of various origins.

Over all patients, color vision testing showed an average color confusion index (CCI) of 1.55 ± 0.60. Tx seemed not to affect color vision, with *P*-values of.79 in the univariable analysis (−0.03, 95% CI [−0.30; 0.39]) and 0.86 in the multivariable analysis (−0.03, 95% CI [−0.03; 0.27]; other independent variables in the model: age, diabetes mellitus, hypertension, aromatase inhibitors).

For the influence of age on color vision, the univariable analysis estimated a change of 0.033 CCI per year (95%CI [0.022; 0.044], *P*-value < .001). The multivariable analysis estimated a change of 0.025 CCI per year (95%CI [0.011 CCI per year; 0.039 CCI per year]; *P*-value of.0004). In the multivariable analysis with independent variables Tx, age, diabetes mellitus, hypertension, and aromatase inhibitors, age was the only factor affecting CCI.

Diabetes mellitus was associated with a change of 0.72 CCI (95% CI [0.17; 1.28]) with a *P*-value of.01 in the univariable analysis.

Hypertension also impacted CCI (estimated change: 0.42 CCI (95% CI [0.17;0.67]; *P*-value = 0.001)) in the univariable analysis.

### Retinal thickness measured by optical coherence tomography

Retinal integrity is crucial for clear vision. Retinal edemas cause an increase in thickness and can be causes by several diseases such as age related macular degeneration or diabetes leading to decreased and blurred central vision. Retinal atrophy and thus thinning appears in advanced glaucoma and also during systemic degenerative or inflammatory diseases causing visual deterioration and visual field defects.

The average central retinal thickness in both eyes was 233.2 ± 22.8 μm. Tx seems to have no relevant effect on retinal thickness, with a *P*-value of.56 in the univariable analysis (−3.53 µm, 95% CI [−15.49 µm; 8.43 µm]) and 0.70 in the multivariable analysis (−2.28 µm, 95% CI [−13.99 µm; 9.43 µm]; other independent variable in the model: age).

Age seems to affect retinal thickness. The univariable analysis showed a decrease of -0.54 μm per year (CI −-0.96 μm; −0.12 μm]; *P* < .001). The multivariable analysis also found a decrease of −0.53 μm per year (CI [−0.96 μm; −0.11 μm]; *P* < .02]; another independent variable in the model: Tx).

Chemotherapy appeared to relevantly impact central retinal thickness in the univariable analysis, with an estimated increase of 11.01 μm. However, due to a wide confidence interval [3.12 μm; 18.90 μm], this estimate is imprecise. The variable was excluded in the multivariable analysis due to this imprecision.

## Discussion

To the best of our knowledge, this is the first study which shows an influence of the duration of tamoxifen therapy on 2 additional ophthalmological side effects, beyond those already known: an increased loss of variance in visual field examination and a notable decrease in corneal thickness as therapy duration extends. These 2 factors may contribute to the development of glaucoma, which can lead to blindness if left untreated. Tamoxifen therapy, a common treatment for ER+ breast cancer, has been associated with several ocular side effects due to the presence of ER+ tissue within the eye.^10^ These side effects include dry eye,^8,13^ reversible acute retinopathy with macular edema, swelling of the optic nerve head and retinal bleeding^14^ and irreversible chronic tamoxifen retinopathy with preretinal crystalline deposits with macular edema,^5,15^ as well as cataract,^6^ keratopathy,^7^ and optic neuritis. We did not detect such side effects, which may be due to the relatively short mean tamoxifen intake as the risk for retinal side effects is known to increase with therapy duration and thus the cumulative tamoxifen dosage.

However, the potential connection between tamoxifen and glaucoma-related changes, particularly loss variance deterioration, has not been explored in detail prior to this study.

Glaucoma diagnosis involves detecting characteristic optic nerve changes along with correlating visual field defects. While Mean defect is often used to measure visual field deterioration, it cannot differentiate between localized or diffuse defects. Loss variance on the other hand, provides a more refined measurement, capturing the homogeneity of defects and detecting changes at an earlier stage.^16^ In fact, even in the early glaucoma stages a decline in loss variance is observed making it a more sensitive marker for detecting early glaucomatous damage compared to Mean defect.^17^

In the present study, we found a clear correlation between the cumulative tamoxifen dose and a loss variance deterioration, suggesting that prolonged tamoxifen use may cause or exacerbate glaucomatous damage.

It is important to note, however, that visual field-testing results can be influenced by factors such as patient compliance, experience with the test, age, and concentration levels. These confounders should be carefully accounted for in future studies to ensure accurate assessment and early detection of ocular side effects associated with tamoxifen therapy. Moreover, an analysis of the retinal nerve fiber layer thickness at the optic nerve head should be conducted.

Our study revealed a novel finding: a correlation between the cumulative tamoxifen dose and a reduction in corneal thickness, a phenomenon that, to our knowledge, has not been previously associated with tamoxifen use.

Interestingly, previous research has linked corneal thickness to hormonal fluctuation, particularly during the female menstrual cycle.^18,19^ The cornea tends to be thinnest just before ovulation when estradiol levels peak.^20^ However, it remains unclear whether these changes in corneal thickness result directly from estrogen’s effect on the cornea or are mediated indirectly through its influence on the renin-angiotensin-aldosterone system.^20^ Similarly, it is uncertain whether tamoxifen’s impact on corneal thickness arises from a direct agonistic action on estrogen receptors, which may increase estrogen levels or through an indirect effect on the renin-angiotensin-aldosterone system.

Corneal thickness is a well-known risk factor for developing glaucoma^21^ making this finding clinically relevant. Additionally, corneal thickness can fluctuate by approximately 40 μm throughout the day,^22^ which introduces variability in measurements. In our study, examinations were conducted at various times of the day, and these diurnal fluctuations should be taken into account in future research to enhance the accuracy of our findings.

The clinically relevant influence of cumulative tamoxifen dose on corneal thickness has practical implications for patients undergoing tamoxifen therapy, particularly those with glaucoma. A thinner cornea can lead to inaccurately low intraocular pressure measurements, potentially delaying the diagnosis and management of glaucoma. Therefore, regular monitoring of corneal thickness and intraocular pressure is critical during follow-up visits for these patients.

It is worth noting, however, that a large-scale study by Panganini-Hill and Clark in 2000 did not find a significant association between tamoxifen therapy and the development of glaucoma.^6^ This highlights the complexity of tamoxifen’s effect on the eye and underscores the need for further research to reconcile these findings.

### Aromatase inhibitor therapy

Tamoxifen was traditionally regarded as the gold standard for treating ER+ breast cancer, including in postmenopausal patients. However, more recent research has shown that aromatase inhibitors are both more effective and safer leading to their widespread use in postmenopausal patients today.^23^ While aromatase inhibitors are beneficial, they are also associated with ocular side effects including dry eye disease and retinal changes such as retinal bleeding, macular edema, uveitis, central artery occlusion, and vitreoretinal traction.^12,24^ Current findings suggest that these side effects may not be linked to the duration of use or the cumulative dose, as they can manifest early in therapy.^25^

In the present study, we observed a small impact of aromatase inhibitor therapy on the eye. Both visual acuity and contrast sensitivity appear to be adversely affected by the treatment, potentially due to one of the previously mentioned side effects. Given the limited number of patients on aromatase inhibitors in this study, further research with a larger study population and more detailed tracking of the treatment (which aromatase inhibitor, duration, etc.) is recommended.

### Study limitations

The following variables were not further evaluated due to insufficient data: alcohol consumption, amblyopia, antibody therapy, glaucoma, GnRH therapy, and hyperthyroidism. A multivariable analysis was not performed in this case because there were too many non-evaluable variables. Studies on larger patient cohorts are required to further evaluate these parameters.

As a glaucoma diagnosis requires a proven optic nerve damage with concomitant visual field defects this study cannot answer the question if the observed changes in corneal thickness and visual field were clearly related to glaucoma or if the patients developed glaucoma later on. As glaucoma develops over years or decades long-term studies are required. Therefore, the findings of this study are not sufficient to justify an interruption or termination of tamoxifen treatment.

## Conclusion

In the present study, a potentially clinically relevant influence of the cumulative tamoxifen dose on changes in loss of variance and corneal thickness was seen. These changes may represent risk factors or early alterations due to glaucomatous damage. Therefore, patients undergoing tamoxifen therapy should be carefully screened for a deterioration of any pre-existing glaucoma or the development of glaucoma.

Further large-scale studies that include patients with longer treatment duration seem advisable. These should incorporate OCT scans with retinal segmentation, as this method can also capture early changes.

## Data Availability

The data that support the findings of this study are available from the corresponding author KS, upon reasonable request.
